# Association of physical activity on body composition, cardiometabolic risk factors, and prevalence of cardiovascular disease in the Korean population (from the fifth Korea national health and nutrition examination survey, 2008–2011)

**DOI:** 10.1186/s12889-017-4126-x

**Published:** 2017-03-21

**Authors:** Gwang-Sil Kim, Eui Im, Ji-Hyuck Rhee

**Affiliations:** 1 Division of Cardiology, Department of Internal Medicine, Sanggye Paik Hospital, Inje University College of Medicine, Seoul, Republic of Korea; 2 Division of Cardiology, Department of Internal Medicine, Yongin Severance Hospital, Yonsei University College of Medicine, Yongin, Republic of Korea; 3 Division of Cardiology, Yongin Severance Hospital, Yonsei University College of Medicine, 225 Geumhak-ro, Cheoin-gu, Yongin, 17046 South Korea

**Keywords:** Cardiovascular disease, Obesity, Sarcopenia, Physical activity

## Abstract

**Background:**

Data regarding associations among physical activity (PA) level, body composition, and prevalence of cardiovascular diseases in Asian populations are rare.

**Methods:**

The International Physical Activity Questionnaire (IPAQ) was utilized to estimate PA levels and analyze the association of PA level with various body composition parameters and the prevalence of cardiovascular diseases by using data from the Korean National Health and Nutrition Examination Survey from 2008 to 2011.

**Results:**

Moderate and high PA levels were associated with lower prevalence of hypertension and diabetes mellitus, and lower concentrations of serum ferritin, parathyroid hormone, and alkaline phosphatase. Sarcopenia (low vs. moderate vs. high PA group: 14.3% vs. 10.5% vs. 7.3%, *p* = 0.001), underweight (5.7% vs. 4.9% vs. 3.5%, *p* = 0.001), and central obesity (7.8% vs. 6.9% vs. 6.3%, *p* = 0.002) were more often observed in the low PA group. The prevalence rates of cardiovascular diseases were lower in the moderate (odds ratio [OR], 0.822; 95% confidence interval [CI], 0.737–0.916; *p* = 0.001) and high activity groups (OR, 0.663; 95% CI, 0.589–0.748; *p* = 0.001) than in the low activity group, even after adjusting for age, sex, smoking, underlying disease, and general or abdominal obesity and muscle mass.

**Conclusion:**

Regular physical activity was associated with a low prevalence of cardiovascular diseases (stroke, myocardial infarction, stable angina, and chronic renal disease), which was independent of body composition and conventional risk factors in the Korean population, with a positive dose-response relationship.

## Background

Cardiovascular disease (CVD) is the leading cause of death and disability worldwide. According to World Health Organization global estimates of mortality and burden of disease, 30% of all global deaths were associated with CVD [1, 2]. Besides increasing mortality, CVD leads to substantial health-care expenditures and decline in economic growth due to decreased work activity and permanent disability [3]. CVD is a multifactorial disease process influenced by various biological and behavioral characteristics. Smoking cessation, physical activity (PA), weight reduction, and healthy diet are the major components of an effective CVD prevention program [4]. These lifestyle modifications are a proven cost-effective strategy for both primary and secondary prevention of CVD [5–8].

Regular PA clearly decreases the risk of CVD at the same magnitude as that of smoking cessation [9–11]. Previous reports documented that the median risk reduction for CVD was about 40% when comparing between most and least active subjects. These results persisted even after adjusting for traditional cardiovascular risk factors such as hypertension (HTN), dyslipidemia, and diabetes mellitus (DM) [12, 13]. Based on this evidence, the federal government issued its first-ever PA guidelines for Americans in 2008 [14].

However, relatively few studies have examined the relationship between PA level and coronary heart disease/CVD risk in nonwhite populations [15]. Moreover, only few studies have simultaneously demonstrated the association among PA level; clinical measures of body composition such as waist circumference (WC), muscle mass, or body mass index (BMI); and the prevalence of CVD.

Therefore, this study was conducted to assess the relationship of PA level as defined by the International Physical Activity Questionnaire-short form (IPAQ-SF) [16] with body composition and prevalence of CVD by analyzing data from the fifth Korean National Health and Nutrition Examination Survey (KNHANES) conducted from 2008 to 2011.

## Methods

### Study population

In this study, data collected from 2008 to 2011 through the KNHANES were used. The target population of the KNHANES included all non-institutionalized Korean civilians of at least 1 year of age. A stratified multi-stage probability sampling design was used. The subjects were selected from sampling units predicated on geographical area, sex, and age, determined by using household registries. Each population group was assigned a weighted value based on geographical and demographic characteristics to allow estimates to be calculated for the entirety of the Korean population. KNHANES is composed of three components surveys: a health interview, heath examination and nutrition survey. The health interview and health examination are performed by trained medical staff and interviewers at the mobile examination center. One week after the health interview and health examination surveys, dieticians visit the home of participants for the nutrition survey. The surveys collect detailed information on socioeconomic status, health behaviours, quality of life, healthcare utilization, anthropometric measures, biochemical profiles using fasting blood serum and urine, measures for dental health, vision, hearing and bone density, X-ray test results, food intake and dietary behavior. The overall response rate was 76.1% for the heath interview and examination survey and 82.4% for the nutrition survey. KNHANES data are valuable sources for monitoring changes in risk factors and diseases and identifying target groups in need of interventions. Korea Centers for Disease Control and Prevention (KCDC) and related academic societies have managed external quality control programmes for all steps (including survey administration, data collection, laboratory analysis and data processing) as well as internal quality assurance and control procedures [17]. The questionnaires used in this study included questions that addressed the demographic, socioeconomic, dietary, and medical history of each respondent [18]. After excluding 9882 subjects younger than 20 years of age and 1777 subjects without information on PA, 26,294 subjects consisting of 11,213 male subjects (42.6%) and 15,081 female subjects (57.4%) were included in our final analysis. The Korea Centers for Disease Control and Prevention (KCDC) Institutional Review Board approved the survey protocol, and all participants provided written informed consent. However, this study did not require any ethics approval, because the KNHANES data are publicly available.

### Definition of PA

The IPAQ-SF is used to estimate the overall PA level of an individual in metabolic equivalent (MET)-min/week by determining the duration (in minutes) and number of days (in 1 week) of engagement in three specific types of activity (walking, moderate-intensity activities, and high-intensity activities) across a comprehensive set of domains (leisure time, work-related and transport-related physical activities, and domestic and gardening activities) in the past 7 days. The IPAQ-SF has acceptable reliability and validity [16, 19] in previous studies. Walking fast, carrying light loads, bicycling at a regular pace, golf or tennis are including in moderate intensity activities and heavy lifting (more than 20 kg), digging, aerobics, basketball, swimming or fast bicycling are including in high-intensity activities. MET is a unit that is used to estimate the amount of oxygen used by the body during PA. MET-min/week is computed by multiplying the MET score of an activity (3.3 for walking, 4.0 for moderate-intensity activities, and 8.0 for vigorous intensity activities) to the minutes and days (or sessions) of engagement. Respondents were classified according to three PA levels, namely low, moderate, or high, according to the cutoff total MET-min/week in each category [20] as follows:

- Category 1 Low
  Individuals who did not meet the criteria for Categories 2 (moderately active) and 3 (highly active) were considered to have low PA levels or as physically inactive.
- Category 2 Moderate
  - Three or more days of vigorous-intensity activities of at least 20 min/day;
  - Five or more days of moderate-intensity activities and/or walking of at least 30 min/day; or
  - Five or more days of any combination of walking, moderate-intensity activities, and vigorous-intensity activities that could attain a minimum total PA of at least 600 MET-min/week.
- Category 3 High
  - Vigorous-intensity activities for at least 3 days that could attain a minimum total PA of at least 1500 MET-min/week; or
  - Seven or more days of any combination of walking, moderate-intensity activities, or vigorous-intensity activities that could attain a minimum total PA of at least 3000 MET-min/week.

### Definition of sarcopenia, obesity, and metabolic syndrome

Appendicular skeletal muscle mass (ASM) was measured by using dual-energy X-ray absorptiometry (QDR 4500A; Hologic Inc.). ASM (in kilograms) was defined as the sum of the lean soft tissue masses of the arms and legs [21]. To define sarcopenia, we assessed ASM as a percentage of body weight (ASM/weight [Wt.]), which was modified from the method used in the study by Janssen et al [22], and defined as <2 SD below the sex-specific mean for a young reference group from the data sets of the 2008 and 2011 KNHANES (960 male subjects and 1240 female subjects, 20–30 years old). The cutoff point for sarcopenia was 29.0% for male subjects and 22.9% for female subjects.

The International Obesity Task Force (IOTF) and World Health Organization (WHO) regional office for the Western Pacific region recommend defining obesity in Asians as a BMI of ≥25 kg/m^2^. Subsequently, the Korean Society for the Study of Obesity (KSSO) adopted this definition [23]. Thus, subjects were classified as obese if their BMIs were ≥25 kg/m^2^ according to the standards of the IOTF, WHO, and KSSO.

The modified National Cholesterol Education Program’s Adult Treatment Panel III (NCEP ATP III) Asian criteria for metabolic syndrome was used, requiring 3 or more of the following criteria: WC, ≥90 cm for male subjects and ≥85 cm for female subjects [24]; triglyceride level, ≥150 mg/dl; high-density lipoprotein (HDL)-cholesterol level, ≤40 mg/dl for male subjects and ≤50 mg/dl for female subjects; blood pressure, ≥130/85 mm Hg; and fasting glucose level, ≥100 mg/dl.

### Definition of CVD

CVD was defined as previous cardiovascular events such as angina pectoris, myocardial infarction, and stroke [25]. We also included chronic renal disease, which was defined as chronic kidney disease stage ≥3 (glomerular filtration rate [GFR] of ≥60 ml/min/1.73 m^2^) in CVD [26, 27]. GFR was estimated by using the Modification of Diet in Renal Disease (MDRD) equation.

### Statistical analyses

All data were expressed as number and percentage (%) or mean ± standard deviation. Continuous variables were compared by using an independent *t* test or analysis of variance. Categorical variables were compared by using the chi-square test or Fisher exact test. The odds ratio (OR) for stroke, myocardial infarction, angina pectoris, and chronic renal disease according to physical groups were calculated by using logistic regression. The multivariable-adjusted model was adjusted for known confounding factors such as age, sex, hypertension, DM, and smoking. *P* values of <0.05 were considered statistically significant. The Statistical Package for the Social Sciences (SPSS, version 19; SPSS, Chicago, IL, USA) was used for these analyses.

## Results

Table 1 shows the baseline characteristics of the participants according to their activity levels. Low, moderate, and high activity groups comprised 20.2, 45.6, and 34.2% of the study population, respectively. Older and female subjects were more prevalent in the low activity group. Resting heart rate was lower in the high activity group than in low or moderate activity group. Serum concentrations of fasting glucose (low > moderate = high activity group, *p* = 0.001), alkaline phosphatase (low > moderate = high activity group, *p* = 0.001), parathyroid hormone (low > moderate > high activity group, *p* = 0.001), ferritin (low > moderate > high activity group, *p* = 0.001) were highest, but vitamin D level was lowest in the low activity group. The associations between clinical measures of body composition and PA are summarized in Table 2. ASM was higher in the high activity group (high > moderate = low activity group, *p* = 0.001). Obesity measure according to BMI did not differ between the groups, but sarcopenia had an inverse relationship with PA level (OR in moderate activity = 0.744, OR in high activity = 0.516, both compared with low activity group, *p* = 0.001). Each metabolic syndrome component was also associated with PA level, and the risk of metabolic syndrome was higher in the low activity group.

**Table 1:** Subject characteristics according to physical activity levels

	Low (*n* = 5309) (20.2%)	Moderate (*n* = 11,997) (45.6%)	High (*n* = 8988) (34.2%)	*P*-value
Age (years)	^a^53 ± 17	^b^49 ± 16	^b^49 ± 15	0.001
Male	1951 (36.7)	4651 (38.8)	4611 (51.3)	0.001
Hypertension	2099 (39.5)	4155 (34.6)	3142 (35.0)	0.001
Diabetes mellitus	669 (12.6)	1302 (10.9)	1074 (11.9)	0.002
Dyslipidemia	488 (9.2)	1168 (9.7)	821 (9.1)	0.283
Current smoker	1573 (29.6)	3471 (28.9)	3431 (38.1)	0.001
Systolic blood pressure (mm Hg)	^a^120 ± 18	^b^118 ± 18	^c^119 ± 17	0.001
Diastolic blood pressure (mm Hg)	^a^76 ± 11	^b^76 ± 11	^b^77 ± 11	0.001
Resting heart rate	^a^71 ± 10	^a^70 ± 9	^b^69 ± 9	0.001
Hemoglobin (mg/dl)	^a^13.7 ± 1.6	^a^13.8 ± 1.6	^b^14.1 ± 1.6	0.002
Creatinine (mg/dl)	0.8 ± 0.3	0.8 ± 0.2	0.8 ± 0.2	0.323
Fasting glucose (mg/dl)	^a^99.1 ± 26.9	^b^97.5 ± 22.4	^b^97.9 ± 22.8	0.001
25 (OH) vitamin D_3_ (ng/mL)	^a^17.5 ± 6.5	^b^17.9 ± 6.5	^c^19.5 ± 7.0	0.001
PTH (pg/ml)	^a^71.3 ± 37.1	^b^67.5 ± 29.6	^c^65.2 ± 26.3	0.001
Ferritin (ng/mL)	^a^82.7 ± 104.8	^b^81.2 ± 110.4	^c^80.4 ± 123.7	0.001
ALP (U/L)	^a^231.3 ± 75.9	^b^223.4 ± 76.6	^b^224.8 ± 69.3	0.001
Triglyceride (mg/dl)	^a^137.3 ± 106.5	^b^133.4 ± 105.1	^b^134.0 ± 118.0	0.101
HDL-cholesterol (mg/dl)	^a^51.5 ± 12.5	^b^52.3 ± 12.7	^b^52.7 ± 13.0	0.004

*ALP* alkaline phosphatase, *HDL* high-density lipoprotein, *25(OH)D* 25-dihydroxyvitamin D; PTH = parathyroid hormone

Values were represented as mean ± standard deviation, or n (%)

^a^, ^b^, ^c^were designated according to post hoc analysis. Same marker was used to represent no statistically significant difference

**Table 2:** Body composition and metabolic syndrome components according to physical activity level

	LOW (*n* = 5309) (20.2%)	Moderate (*n* = 11,997) (45.6%)	High (*n* = 8988) (34.2%)	*p* value
ASM (kg)	17.1 ± 4.6	17.7 ± 4.7	19.2 ± 4.9	0.001
ASM/weight (%)	18.9 ± 13.7	20.2 ± 13.5	21.7 ± 13.9	0.001
Sarcopenia	509 (14.3)	892 (10.5)	477 (7.3)	0.001
BMI > 25 kg/m^2^	260 (4.9)	539 (4.5)	441 (4.9)	0.289
BMI < 18.5 kg/m^2^	301 (5.7)	587 (4.9)	311 (3.5)	0.001
Sarcopenic obesity	157 (4.4)	321 (3.8)	252 (3.9)	0.274
Metabolic syndrome	428 (8.1)	846 (7.1)	645 (7.2)	0.054
^a^Central obesity	413 (7.8)	823 (6.9)	564 (6.3)	0.002
High triglyceride	1515 (30.6)	3292 (28.7)	2453 (28.1)	0.006
Low HDL	1667 (33.7)	3558 (31.0)	2324 (26.6)	0.001
Hypertension	2099 (39.5)	4155 (34.6)	3142 (35.0)	0.001
Diabetes mellitus	669 (12.6)	1302 (10.9)	1074 (11.9)	0.002

*ASM* appendicular skeletal muscle mass, *BMI* body mass index, *HDL* high-density lipoprotein

^a^Waist circumference of ≥90 cm for male subjects and ≥85 cm for female subjects

The ORs for the prevalence of CVD, as calculated by using logistic regression analyses, are shown in Table 3. On the univariate analysis, the prevalence of CVD was significantly lower in the moderate (OR = 0.722, *p* = 0.001) or high activity group (OR = 0.610, *p* = 0.001) than in the low activity group. This difference in prevalence remained significant after adjustment for conventional risk factors of CVD, including age, HTN, DM, smoking, and male sex. Besides PA level, sarcopenia, BMI, and central obesity were associated with the prevalence of CVD (Table 3).

**Table 3:** Odds ratio of cardiovascular disease (MI, stable angina, stroke, and chronic renal disease) according to physical activity, body composition, and metabolic syndrome component

	Non-adjusted		^b^Adjusted	
	Odds ratio (95% confidence interval)	*p* value	Odds ratio (95% confidence interval)	*p* value
Activity				
Low	1			
Moderate	0.72 (0.65–0.80)	0.001	0.82 (0.74–0.92)	0.001
High	0.61 (0.55–0.68)	0.001	0.66 (0.59–0.75)	0.001
Sarcopenia	2.17 (1.95–2.43)	0.001	1.63 (1.45–1.84)	0.001
BMI > 25 kg/m^2^	1.32 (1.10–1.57)	0.003	1.30 (1.08–1.57)	0.005
^a^Central obesity	2.40 (2.11–2.72)	0.001	1.82 (1.59–2.08)	0.001
High triglyceride	1.58 (1.45–1.73)	0.001	1.21 (1.10–1.33)	0.001
Low HDL	1.64 (1.50–1.79)	0.001	1.58 (1.44–1.74)	0.001

*BMI* body mass index, *HDL* high-density lipoprotein, *MI* myocardial infarction

^a^Waist circumference of ≥90 cm for male subjects and ≥85 cm for female subjects

^b^Adjusted variables: age of >65 years, hypertension, diabetes mellitus, sex, and smoking

**Fig. 1:**
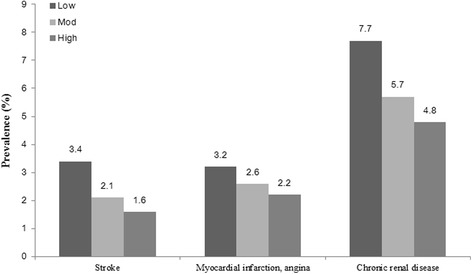
Prevalence of cardiovascular disease according to physical activityᅟ

**Fig. 2:**
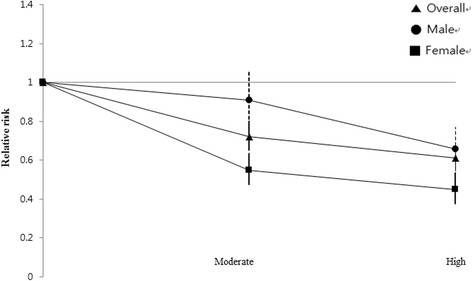
Relative risk of CVD according to physical activity

Then, we performed a multivariate logistic analysis in order to adjust for the body composition parameters and metabolic risk components, which were regarded as independent risk factors of CVD. In the comparison between high or moderate activity group and the low activity group, the odds ratio was still significant after adjustment for each variable, except for sarcopenia, which showed a difference only between the high and low activity groups (Table 4). Figure 1 shows that each CVD component including stroke, coronary heart disease and chronic renal failure also had an inverse relationship with PA level, and Fig. 2 demonstrates a dose-response relationship between PA level and CVD prevalence and it is more prominent in female group.

**Table 4:** Association of physical activity level with CVD after adjustment for data sets of potential mediators

	Low	Moderate	*p* value	High	*p* value
Age of >65 years and sex	1.00	0.82 (0.71–0.95)	0.001	0.678 (0.58–0.80)	0.001
Basic model^a^	1.00	0.82 (0.74-0.92)	0.029	0.66 (0.59–0.75)	0.001
Basic model plus each set of risk factors below					
BMI > 25 kg/m^2^	1.00	0.82 (0.71–0.95)	0.025	0.67 (0.57–0.79)	0.001
Sarcopenia	1.00	0.87 (0.73–1.05)	0.287	0.75 (0.61–0.92)	0.005
Central obesity	1.00	0.83 (0.71–0.96)	0.031	0.68 (0.57–0.80)	0.014
High triglyceride	1.00	0.81 (0.70–0.95)	0.029	0.70 (0.59–0.83)	0.001
Low HDL	1.00	0.81 (0.70–0.95)	0.029	0.71 (0.60–0.84)	0.001

*BMI* body mass index, *CVD* cardiovascular disease

^a^Age, sex, smoking, hypertension, and diabetes mellitus

## Discussion

The main findings of this study were as follows: 1) the prevalence rates of HTN, DM, and resting heart rate were lower in the moderate or high activity group than in the low activity group; 2) the moderate or high activity group had more balanced body composition and lower risk of metabolic syndrome components, including HDL and TG level; 3) PA was associated with a lower prevalence of CVD, and this association remained after adjustment for the potential confounding factors (age, sex, smoking, HTN, and DM), and body composition parameters and metabolic syndrome components (muscle mass, BMI, central obesity, low HDL level, and high triglyceride level).

In 1953, Morris et al. [28] first reported the association between PA and protection against CVD. Since then, many other studies have been conducted and have yielded similar results. The PA guidelines for Americans that were published in 2008 recommend that 150 min/week of moderate-intensity aerobic PA or 75 min/week of vigorous-intensity aerobic PA is associated with protection from CVD [14]. Although the precise mechanisms of the reduced risk of CVD associated with PA are not well understood, reductions in the traditional risk factors, including blood pressure, BMI, glucose abnormalities [16, 29], and the chronic anti-inflammatory effect, mediated by PA were proposed as possible mechanisms [13]. However, most prospective cohort studies that used self-reported data on PA to examine its association with CVD risk were published in Western countries [15], and PA and its association with body composition or CVD varies with race and lifestyle. In accordance with previous studies, the prevalence rates of HTN and DM and fasting glucose level were higher in the low activity group than in the moderate or high group. Resting heart rate was lower in the high activity group than in the low or moderate group. In previous studies, elevated resting heart rate was associated with metabolic syndrome and poor outcome in patients with heart failure or coronary heart disease [30, 31]. It is interesting that the levels of biomarkers such as vitamin D, serum ferritin, ALP, and PTH differed according to PA level, as shown in Table 1. Vitamin D deficiency and elevated levels of ALP, ferritin, and PTH were identified as predictors of metabolic syndrome or poor prognosis of CVD in previous studies [32–35]. Thus, the favorable effect of PA on the prevalence of CVD might have relevance to these biomarkers, and further prospective studies are needed.

The moderate or high activity group had significantly lower prevalence rates of sarcopenia, underweight, and abdominal obesity, whereas no significant difference was observed in general obesity defined as a BMI of ≥25 kg/m^2^. From these findings, we can infer that regular PA does not simply reduce weight but should be coupled with more balanced body composition and physical fitness, as sarcopenia, central obesity, and underweight were reported as independent risk factors of developing CVD and mortality or poor prognosis in patients with CVD and general obesity [36–41]. Analysis of the association between PA and CVD revealed a positive dose-response relationship, with an OR of 0.822 (95% CI, 0.737–0.916) for the moderate group and 0.663 (0.589–0.748) for the high group. In IPAQ scale, strength trainings such as digging, heavy lifting or fast bicycling are included in moderate to high intensity activities. These activities strengthen and maintain lean muscle mass as well as burning calories. Previous studies reported that muscle mass is associated with cardiovascular risk, prevalence of cardiovascular disease and insulin resistance [42–45]. Thus, in line with previous studies, the results of this study also showed strength trainings assessed by IPAQ scale is associated with physical fitness and lower prevalence of CVD. These risk reduction remained significant even after adjusting for conventional risk factors of CVD, including age, HTN, DM, sex, and smoking. A dose-response relationship was difficult to define because of the different questionnaires used across various studies to assess PA in 1 or more domains of activity (leisure-time, household, occupation, and commuting activity), with most of the studies assessing primarily leisure-time PA [15]. In our study, we used the IPAQ as mentioned earlier, and the classification of PA was similar with the definition in the 2008 American guidelines [14], which defined “moderate” as at least 150 min/week of moderate-intensity activity or 75 min/week of vigorous activity and “high” as 300 min/week of moderate-intensity activity or 150 min/week of vigorous activity. Previous studies that were based on assessments according to the 2008 guidelines reported that the moderate- and high-intensity groups had 20–25% and 30–35% reduction in the risk of CVD [12, 13, 46]. These results approximately equal our data, as the risk reduction rates were 28 and 34% in the moderate and high groups, respectively. Furthermore, the OR was more significant in the female subjects than in the male subjects, as shown in Fig. 2, concurring with the results of previous studies that reported a median risk reduction of 40% in female subjects and 30% in male subjects [47–50]. Thus, the dose-response relationship and prominent effect of PA in female subjects were observed in the Asian population. Considering of these clear relationship with IPAQ scale and physical fitness and clinical outcome, IPAQ could be used as a reliable questionnaire to assess physical activity in patients with high risk of CVD event including patients with diabetes, obesity and metabolic syndrome.

To further understand the association between PA level and CVD, an additional logistic analysis was conducted after combining PA with the conventional risk factors and body composition parameters (Table 4). After adjusting for these factors, the relationship in the moderate or high activity group remained significant. Whether higher PA level mitigate the risk of CVD associated with being overweight or obese is controversial [51–53]. Our results show that PA level has a strong association with the prevalence of CVD, independent of body composition, including both general and central obesities and other conventional risk factors of CVD.

Some limitations should be considered in the interpretation of this study. First, it used a cross-sectional design, which limited the ability to detect causal relationships. Therefore, the cause-and-effect relationships of PA level with cardiometabolic risk factors and prevalence of CVD cannot be precisely inferred, although this study included a representative sample of the general Korean population. Second, this study was based on data obtained by using the IPAQ-short form for PA level. Thus, we could not fully exclude the effects of information bias. Third, although all participants were initially evaluated the heath interview and heath examination at the mobile examination center (bus) which means at least they were able to ambulate, it is impossible to fully exclude the possibility of reverse causation of the association between PA and CVD. It means that our results confined the relationship between the PA and prevalence of CVD not causal relationship of them. Thus, further randomized controlled trials regarding the incidence of CVD according to PA are necessary.

## Conclusions

In conclusion, moderate or high PA level was associated with a low prevalence of CVD (stroke, myocardial infarction, stable angina, and chronic renal disease), which was independent of body composition (general or central obesity, and sarcopenia) and conventional risk factors in the Korean population. Furthermore, the dose-response relationship between PA level and the prevalence of CVD, and the more-prominent effect in female subjects were in line with those in previous studies conducted in the Western population.

## Abbreviations

BMI: Body mass index
CVD: Cardiovascular disease
DM: Diabetes mellitus
HTN: Hypertension
IPAQ: International physical activity questionnaire
KNHANES: Korean national health and nutrition examination survey
MET: Metabolic equivalent
PA: Physical activity
WC: Waist circumference
